# Pellagra: The Lack of Persistence in Its Treatment

**Published:** 1917-05

**Authors:** C. C. Parrish

**Affiliations:** Fort Worth, Texas


### Pellagra: The Lack of Persistence in its Treatment.

BY C. C. PARRISH, M. D., FORT WORTH, TEXAS.

This data was obtained from letters received from a total of 83 doctors; 16 of this number had had no experience, 67 of the total number having experience. This 67 reported a total of 496 cases, with a total of 30 deaths. The average number of cases per man was 7.3 patients, and for convenience I have divided these doctors into two groups: Those that had more than 7.3 patients, and those that had seen less than this number. Twenty-one doctors had seen more than 7.3 patients, and 46 doctors had seen less than 7.3 patients. The twenty-one above the average reported 244 cases, while the 46 below the average reported 148 cases, or 3.7 patients per man. Those above the average had seen 15.5 patients per man. Those above the average left 348 cases unaccounted for, by death or otherwise. Those below the average reported 23 deaths; 41 cases on hand; 86 not accounted for, or 58 per cent not accounted for. Thirty-three doctors still had 131 patients on hand out of 496 cases; 30 had died, leaving 335 cases unaccounted for, or 69 per cent. Those 34 doctors having no patients on hand at time of report had seen 119 cases. The 33 doctors that had 131 on hand had seen a total of 377 cases; deducting 11 deaths that these same doctors reported, it would show 142 cases out of 377 accounted for, with a total of 235 cases out of 377 that they did not account for, or 62 per cent.

Of the doctors having had experience, 19 per cent of them reported 70 per cent of all cases reported, but had only accounted for 38 per cent, leaving 73 per cent of all cases reported unaccounted for.

How many men that treat pellagra keep up with the patient after he or she is relieved? Do you get reports from them from year to year or month to month? Do you know how many of them have return of symptoms after they have had arsenic in some form and get relief? Do you know how long that relief has lasted in each case? If not, you have not the interest in these patients that they have a right to expect you to have when they turn their case over to you. Seventy-five per cent of the cases that I have treated had been given arsenic in some form by some other doctor and had had a recurrence of symptoms and had drifted from one doctor to another, and finally most of them in despair had taken Dr. Balm's Remedy, after which they had given up in disgust. And when I asked them about when they saw their doctor last, the most of them asked me, "Which doctor?" They don't seem to know who their doctor is. In fact, most of them have drifted from one doctor to another until they are lost. No one claims them; no one would claim them as their patient and be proud of them at the time that I see most of them.

It is not always the doctor's fault. At least, if so, it is that he has a virtue to a fault, in that he is too eager to take these cases as he would any ordinary case. We should not be eager to take these cases unless they understand, in the beginning, that they have a chronic case that takes a long time to cure if *their* case can be cured at all and that if they are just going to try us without really giving us the chance, and then go away, as many will do, and say they have tried us and we did them no good. We can't cure these cases in a few weeks or even months, and we may as well tell the patient so in the beginning. Then they won't be disappointed and in a little while run away to their friends or to some other doctor or, worse yet, to some other doctor's friends and say they tried you. Tried you is right—it was a trial; some ordeal, and we don't get thanks for it, if we get paid. But it is our fault, unless we start right in the beginning. Tell them honestly that you can't do them any permanent good, short of a year or two, if at all. Some of them you won't cure, because some of them that look like they should get well may die in a short time, and others that look like they would die in a short time will get well, much to your surprise.

But if we are honest with them, they will know it and will be our friends. Some of my best friends are the families of patients that I have done the least for, but I believe what I told them at the start and told them what I believed, and they knew that I believed it. One thing I don't do—when I have done all that I can for a patient I don't tell them that I have done all that is known to medical science, and that I have done all that can be done; but I tell them I have done all that I know to do; that some other doctor may know something or some method that I don't know, and I tell them that we had better get another doctor. I fear that not enough of us do this. It has happened several times in my experience that the patient had been told by an honest doctor that all had been done that could be done, and I regret to say that this was not true. It is much worse to have the patient take his case out of your hands and take it to a doctor of his own selection than for you to take him to some doctor of your selection. For you not only retain the good will of the patient, but get the good will of the other doctor and I find that to be a valuable asset, both the good will of the doctor and the patient. The other doctor is not half so liable to "knock" you as if you wait until the patient escapes with his case and takes it to some doctor that may not be your friend. We don't any of us know all about pellagra yet, and we are still studying how to handle these cases best; so a little thought along this line may not be amiss, as we perfect our method of dealing with the patient, we more nearly perfect our method of handling the disease.

